# Coronary Injury Score Correlates with Proliferating Cells and Alpha-Smooth Muscle Actin Expression in Stented Porcine Coronary Arteries

**DOI:** 10.1371/journal.pone.0138539

**Published:** 2015-09-18

**Authors:** Vicki J. Swier, Lin Tang, Kristopher D. Krueger, Mohamed M. Radwan, Michael G. Del Core, Devendra K. Agrawal

**Affiliations:** Center for Clinical and Translational Science, Creighton University School of Medicine, Omaha, Nebraska, United States of America; National Jewish Health, UNITED STATES

## Abstract

Neointimal formation and cell proliferation resulting into in-stent restenosis is a major pathophysiological event following the deployment of stents in the coronary arteries. In this study, we assessed the degree of injury, based on damage to internal elastic lamina, media, external elastic lamina, and adventitia following the intravascular stenting, and its relationship with the degree of smooth muscle cell proliferation. We examined the smooth muscle cell proliferation and their phenotype at different levels of stent injury in the coronary arteries of domestic swine fed a normal swine diet. Five weeks after stent implantation, swine with and without stents were euthanized and coronaries were excised. Arteries were embedded in methyl methacrylate and sections were stained with H&E, trichrome, and Movat’s pentachrome. The expression of Ki67, α-smooth muscle actin (SMA), vimentin, and HMGB1 was evaluated by immunofluorescence. There was a positive correlation between percent area stenosis and injury score. The distribution of SMA and vimentin was correlated with the degree of arterial injury such that arteries that had an injury score >2 did not have immunoreactivity to SMA in the neointimal cells near the stent struts, but these neointimal cells were positive for vimentin, suggesting a change in the smooth muscle cell phenotype. The Ki67 and HMGB1 immunoreactivity was highly correlated with the fragmentation of the IEL and injury in the tunica media. Thus, the extent of coronary arterial injury during interventional procedure will dictate the degree of neointimal hyperplasia, in-stent restenosis, and smooth muscle cell phenotype.

## Introduction

The implantation of coronary stents is still a common coronary interventional procedure for patients with coronary artery disease (CAD). In one year, the Healthcare Cost and Utilization Project documented a total of 644, 240 hospital stays that involved the implantation of a cardiac stent [1], which included an aggregate cost of 12 billion dollars to the United States.

Though stent implantation is a useful procedure in patients with CAD, stents may damage the arterial wall. Stent insertion causes the entire artery to stretch, the endothelial layer to be denuded and the plaque to be compressed. Eventually, the internal elastic lamina (IEL) may become fractured, the tunica media depressed, and the adventitia dissected from the artery. Such events promote a local inflammatory response in the injured vessel wall, which is characterized by smooth muscle cell proliferation and migration, and neointimal formation. The neointimal formation may lead to in-stent restenosis, requiring the stent to be replaced in only a few years.

In previous porcine studies, the amount of neointimal thickening [2–3], percent area stenosis [2], and the extent of the inflammatory response [4] were proportional to the degree of arterial injury. The degree of proliferation at individual stent sites was correlated with arterial injury at that site without any systematic evaluation of the phenotype of smooth muscle cells and the underlying mechanisms [2].

Injury to specific arterial structures such as the internal elastic lamina and the tunica media have been proposed to be the instigators of smooth muscle cell proliferation that lead to neointimal formation. In humans, the mean artery injury score was greater in vessels with damaged media than in vessels with just compressed or normal media [5]. Greater neointimal thickness was associated with stunt struts compacting damaged media rather than from struts compacting the arterial plaque.

We have previously reported a significantly greater inflammatory response (an increased intimal area to medial area ratio; and an increased intimal area normalized for injury score) in both stented coronary and iliac vessels compared to their non-stented counterparts [6]. In this article, we compared non-stented to stented porcine coronary arteries to document differential histological responses to arterial injury. We compared the injury score and percent area stenosis data from Krueger et al. [6] to immunostaining of Ki67 (proliferative marker), alpha-smooth muscle actin (contractile smooth muscle marker), vimentin (synthetic smooth muscle marker) and high mobility group box 1 (HMGB1; inflammation marker) in these stented coronary arteries. We combined these parameters to determine the proliferation and smooth muscle phenotype characteristics in stented porcine coronary vessels with differing levels of arterial injury during the interventional procedures.

## Materials and Methods

### Porcine Model

Creighton University’s Institutional Animal Care and Use Committee approved research protocol (0488). Swine were housed and cared for according to NIH and USDA guidelines in the Animal Resource Facility of Creighton University. Domestic pigs were obtained from Jon Swanson Farms (Mamo, NE) and the UNL Animal Research Facility (Lincoln, NE).

All pigs in the study were fed a normal diet (Teklad Miniswine diet 8753, Harlan Laboratories) composed of wheat middlings, ground corn, soybean hulls, dehulled soybean meal, dehydrated alfalfa meal, dicalcium phosphate, and soybean oil. Within the diet, 57% of the kilocalories were from carbohydrates, 28% of the kilocalories from protein, and 15% of the kilocalories from fat. Multi-Link (Guidant, Temecula, California) stent implantation was performed in either the left anterior descending artery or left circumflex artery in fourteen of the domestic swine following the procedures described in Krueger et al. [6]. Three days before stent implantation, swine were given aspirin (350 mg/day) and ticlopidine (325 mg/day), and these medications were continued until one month after the stent placement. Ketamine (20 mg/kg) and xylazine (4 mg/ml) were given to each animal before intubation and isoflurane was used for anesthesia during the interventional procedure. After carotid cut down, a 6F introducer sheath was placed into the right or left internal carotid artery. Heparin (5000 U) was given to the animal, and the coronary artery was visualized angiographically using a 6F JR4 catheter. Multilink stents (Guidant, Temecula, CA) were placed using balloon dilation that generated a stent to artery ratio of 1.1–1.2:1. After angiography, the catheters were removed, the internal carotid was ligated, and animals were allowed to recover. Five weeks after the stents were implanted, the swine were euthanized with an overdose of barbiturate (100 mg/kg sodium pentobarbital IV). Four other domestic swine in the control group were fed the normal diet but were excluded from the stent implantation surgery.

### Histology

Whole heart was removed and coronary arteries were excised. Right and left coronaries were fixed in 4% paraformaldehyde in phosphate buffered saline overnight at 4°C. The samples were processed with phosphate-buffered saline, alcohol gradients, and xylene. Non-stented vessels from the control swine were embedded in paraffin and 4.5μm sections of the paraffin block were cut and placed on slides.

Stented vessels were incubated in a 1:1 mixture of methyl methacrylate/xylene at 4°C for 1 hour and then embedded in a 1:1 mixture of methyl methacrylate (solution B) overnight at 4°C.

The next day, the stented samples were polymerized in a 2:3 ratio of solution A to solution B (2% benzoyl peroxide in methyl methacrylate and 1% polyethylene glycol-400 to 0.5% N,N-dimethyl-p-toluidine in butyl methacrylate). Samples were allowed to polymerize overnight at -22°C. Tissue blocks were cut and shaped with a band saw and rough grinder [6].

Sections were cut with a tungsten carbide knife (Leica, Germany) within a Leica RM2265 rotary microtome (Leica, Germany). These sections were allowed to rehydrate in ethanol and attached to slides with Haupt’s solution. The slides were then compressed in a slide press and baked in a 70°C oven overnight.

Slides were stained with hematoxylin and eosin (H&E) and Trichrome and Movat’s Pentachrome stain. The expression of Ki67+ cells, alpha-smooth muscle actin, vimentin, and HMGB1 was evaluated by immunofluorescence (IF).

### Histological Staining

Tissue sections were routinely stained with hematoxylin and eosin; trichrome stained with a Thermo Scientific* Richard-Allan Scientific* Masson Trichrome Kit (Kalamazoo, MI) per the manufacturers’ instructions, and pentachrome stained with the American MasterTech Russell-Movat’s Pentachrome stain kit (Lodi, CA) per manufacturers’ instructions.

### Immunofluorescence

Tissue sections on the slides were deparaffinized in xylene, rehydrated in ethanol, and rinsed in double-distilled water. Antigen retrieval was at 92C for 20 minutes using LabVision™ HIER Buffer L (Thermo Fisher Scientific Inc), slides were rinsed in 1xPBS, and tissue sections on the slides were incubated in normal goat serum from Vector Laboratories for 2 hours at room temp. Primary antibody from Abcam® (Anti-Ki67 ab15580) or (anti-alpha smooth muscle actin ab7817 and vimentin ab8069) or Santa Cruz biotechnology®, Inc. (HMG-1 sc-26351) was then added overnight at 4°C. On the following day, slides were rinsed in 1xPBS and the secondary antibody (Alexa Fluor® 594-conjugated AffiniPure Goat Anti-Rabbit for Ki67) or (Goat anti-mouse IgG1, Alexa Fluor® 594 for vimentin with Alexa Fluor® 488-conjugated AffiniPure goat anti-rabbit for alpha actin) or (Alexa Fluor® 594-conjugated AffiniPure Donkey anti-goat for HMG-1) was incubated on the slides for 2 hours at room temp. Slides were then rinsed in 1xPBS and Vector laboratories DAPI mounting medium was added to each slide. Tissue sections were viewed with an Olympus BX-51 epi-fluorescent microscope and images were photographed with an Olympus DP71 camera. These images were captured using Olympus cellSens Standard 1.11 software (Waltham, MA).

### Morphological and Data analysis

The morphological analysis follows that of Krueger et al. [6] regarding the measurement of the lumen area and area within the internal elastic lamina (IEL) to determine percent area stenosis. Stented artery sections were assigned a mean injury score as described in Schwartz et al. [2]. The percent area stenosis for stented versus non-stented coronary arteries was compared with an unpaired t-test and possible correlations between percent area stenosis and injury score were tested with linear regression and Pearson r using GraphPad Prism 6 (La Jolla, CA). All data are presented as mean ± SEM (N = 3 in each injury score group). Statistically significant differences were determined at p < 0.05.

## Results

### Comparison of Morphometric Analysis

**Fig 1:**
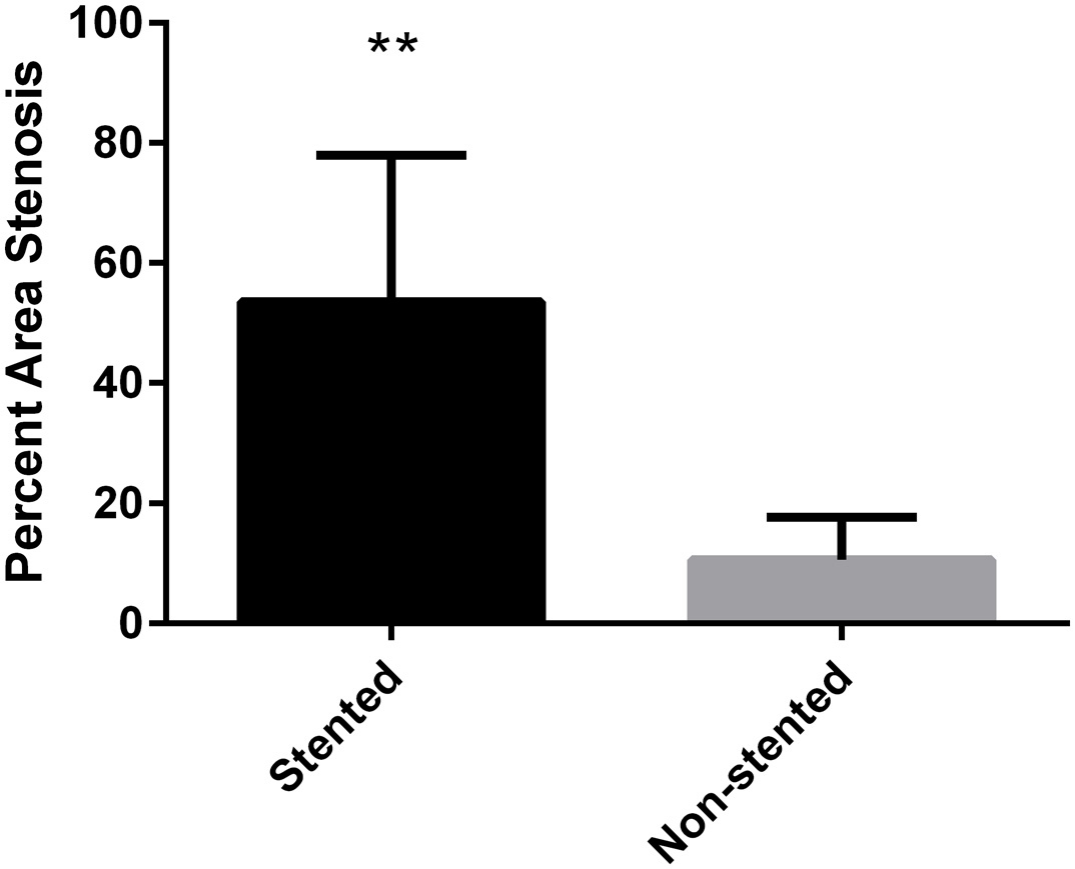
Percent area stenosis between non-stented and stented swine coronary arteries. The mean percent area stenosis of stented coronaries was 53.52% (±7.36) and for non-stented swine coronaries was 10.64% (±3.52); N = 15; **p = 0.004.

The percent area stenosis was statistically greater in the stented swine coronaries compared to the non-stented swine coronaries (p = 0.004) with a mean percent area stenosis of 53.52% (±7.36) in the stented swine arteries and 10.64% (±3.52) in the non-stented coronary arteries (Fig 1).

**Fig 2:**
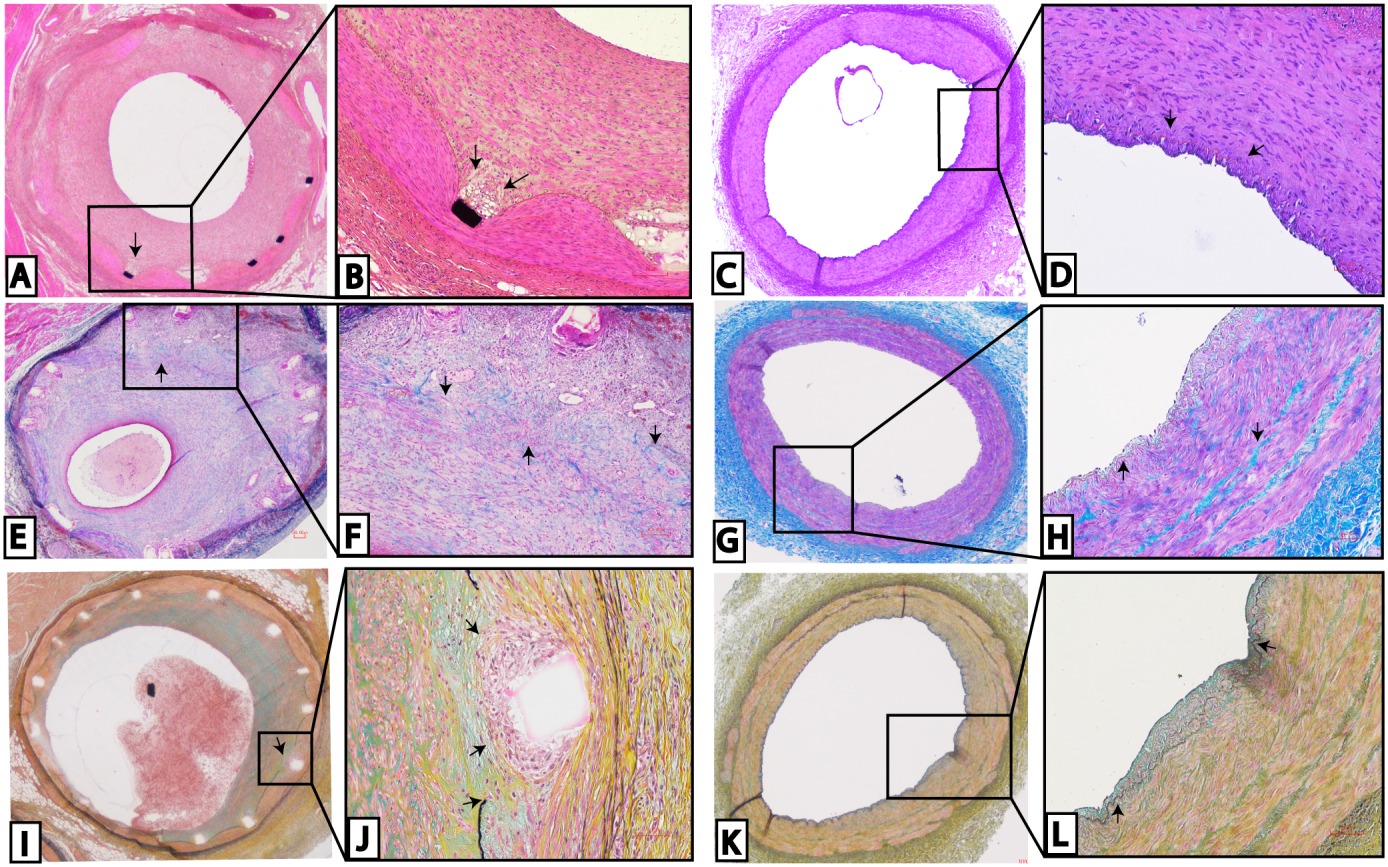
Histology of stented versus non-stented swine coronary arteries. In the H&E staining of stented (A-B) and non-stented arteries (C-D), the necrosis around the stent struts in (B) and the lack of neointima lesions in (D) are shown. Trichrome staining shows the histology of stented (E-F) versus non-stented (G-H) swine coronary arteries. Collagen deposition was found throughout the neointima in stented coronary arteries (F) and in the media of non-stented coronary arteries (H). Movat’s pentachrome staining is shown in the stented (I-J) and non-stented (K-L) swine coronary arteries. The fragmentation of the IEL and the accumulation of inflammatory cells around the stent struts were noted (J). There is a small intimal legion in the non-stented arteries that is mainly composed of proteoglycans (L). These are representative histological slides from N = 3 in each injury score group. Scale bar = 100 μm.

The size of the neointima region in the stented coronary arteries reflected this degree of stenosis (Fig 2A) as there was an expansion of intima that crossed the boundary of the internal elastic lamina in the stented coronary arteries; this was not present in the non-stented coronary arteries (Fig 2C and 2D). The tunica media was compressed in the stented coronaries (Fig 2A and 2B) and the lack of eosinophilic structures in the neointima shows the reduction in cellular cytoplasm compared to eosinophilic-rich tunica media. The lack of any staining around some stent struts may indicate necrotic tissue (Fig 2B). In the trichrome staining, all vessels showed deposition of collagen, as evidenced by blue staining within the neointima of the stented coronary arteries (Fig 2E and 2F) and blue staining in the tunica media of the non-stented coronary arteries (Fig 2G and 2H). There were patches of collagenous fibers within the neointima, closer to the stent struts than to the lumen (Fig 2E and 2F), and these fibers may be proteoglycan rich as they stained green (Fig 2I and 2J) after Movat’s pentachrome staining. In some of the stented coronary vessels there is also a fragmentation of the internal elastic lamina and the presence of inflammatory cells in areas near the stent struts (Fig 2J). Proteoglycans were also present in the media of the non-stented coronaries and within the small neointima area (Fig 2K and 2L).

**Fig 3:**
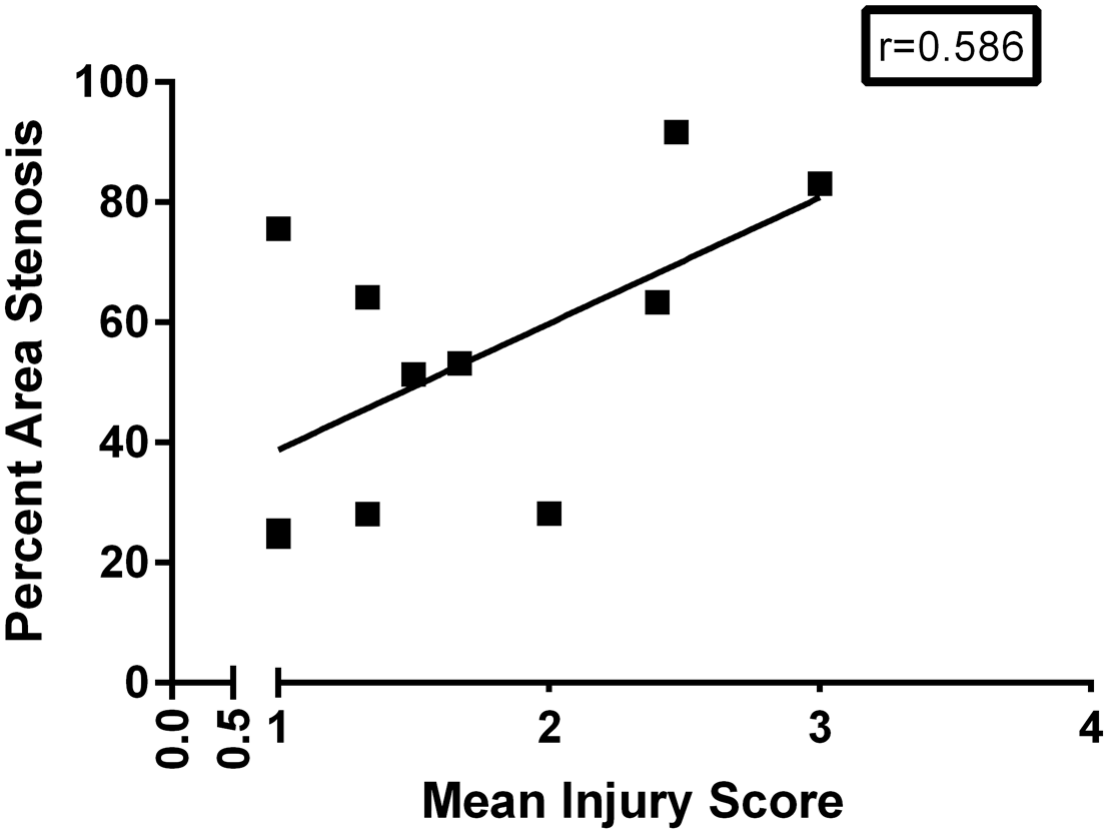
Scatterplot of percent area stenosis versus mean injury score for stented swine coronary arteries. A positive correlation was observed (r = 0.586 p = 0.05), arterial injury increased with increase in percent area stenosis. N = 11.

Injury score was not a complete predictor for percent area stenosis as the injury score explained only 34% of the variance in percent area stenosis (R^2^ = 0.344). The scatter plot reflects this since some of the data points did not fit the regression line (Fig 3). Yet, there was a possible trend towards statistical significance (r = 0.586 p = 0.05) regarding the correlation between percent area stenosis and injury score. A positive correlation may exist between percent area stenosis and injury score (as arterial injury increased, so did percent area stenosis).

### Immunofluorescence

**Fig 4:**
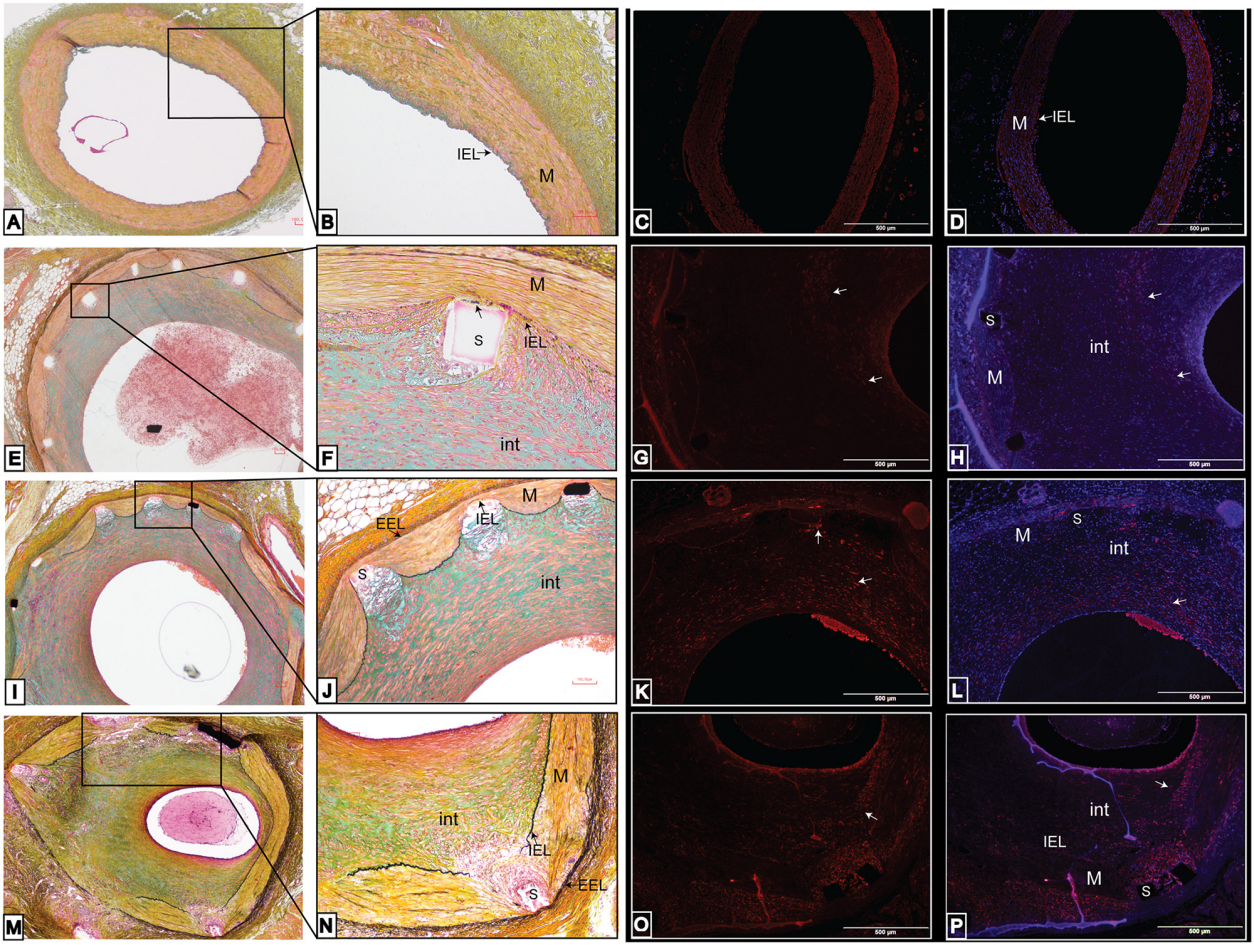
Comparison of Movat’s pentachrome staining in swine coronary arteries to the immunostaining of Ki67. Histology is shown in non-stented (A-D) and stented (E-P) swine coronary arteries. There was a lack of immunoreactivity to Ki67 antibody in the non-stented swine coronary arteries (C-D). The pentachrome staining in stented swine coronary arteries with an injury score of less than 1 (E-H) shows the intact IEL and media layer (F). The immunostaining of Ki67 is found solely in the neointimal region (G-H). The pentachrome staining in stented swine coronary arteries with an injury score of 1–1.5 (I-L) shows the fragmented IEL and compressed media (J). The immunostaining of Ki67 is found in the neointima and around the stent struts (K-L). The pentachrome staining of the stented swine coronary arteries with an injury score greater than 2 (M-P) shows the lacerated IEL and media and the compression of the EEL (N). The immunostaining of Ki67 is found in the neointima, media, and greatly expressed in cells around the stent struts (O-P). These are representative histological slides from N = 3 in each injury score group.

The non-stented coronary arteries lacked a substantial neointimal area (Fig 4A and 4B) and the immunostaining to Ki67 was only present in a few cells near the lumen (Fig 4C and 4D). In contrast, the immunostaining to Ki67 varied depending on the degree of arterial injury in the stented coronary arteries. In stented coronary arteries with a mean injury score less than 1, the IEL was complete, and the media was not compressed (Fig 4E and 4F). The immunostaining of Ki67 in these coronaries arteries was only found in the neointimal area, closer to the lumen (Fig 4G and 4H). Only a few cells in the media or near the stent struts had a positive immunoreactivity to Ki67. In stented coronary arteries with a mean injury score that ranged from 1 to 1.5, the IEL was fragmented, the media was compressed, and necrosis was found near the stent struts (Fig 4I and 4J). The immunostaining of Ki67 was found throughout the neointima (more concentrated towards the lumen) and near the stent struts (Fig 4K and 4L).

In stented coronary arteries with a mean injury score greater than 2, the IEL was completely fractured near the stent struts, the media was lacerated, the EEL (external elastic lamina) was compressed, the neointima was enlarged, and inflammatory cells were circumferentially localized around the stent struts (Fig 4M and 4N). The immunostaining of Ki67 was present in the adventitia, densely located around the stent struts, in the neointima, and within cells surrounding the lumen (Fig 4O and 4P).

**Fig 5:**
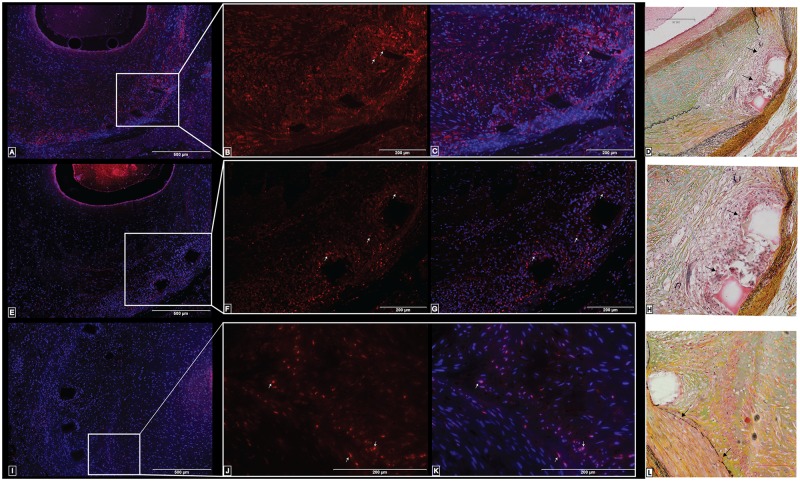
Distribution of HMGB1+ cells and Correlation of Ki67 with HMGB1. Ki67 expression in stented swine coronary artery with an injury score greater than 2 (A-C) shows the concentration of Ki67+ cells around the stent struts (A-C) and the corresponding Movat’s pentachrome stain showing the fragmented IEL and inflamed stent strut (D). HMGB1 expression in cells surrounding the stent struts (E-G) in stented swine coronary artery with an injury score greater than 2 shows that some cells around the stent struts express both HMGB1 and Ki67. HMGB1 expression in smooth muscle cells (I-K) in stented swine coronary artery with an injury score greater than 2 demonstrates the location of the positive smooth muscle cells in relation to the fragmented IEL (L). These are representative histological slides from N = 3 in each injury score group.

In the stented coronary arteries with an injury score greater than 2, the immunostaining of Ki67 was found in cells near the stent struts (Fig 5A–5C) which corresponded to areas that had a completely fractured IEL and an accumulation of inflammatory cells (Fig 5D). The cells within these injured areas were also HMGB1^+^ (Fig 5E–5G), and appear to be undergoing necrosis or apoptosis based on their morphological appearance (Fig 5H). Cells near the IEL were also HMGB1^+^ (Fig 5I–5K). These HMBG1^+^ smooth muscle cells were aligned next to fragmented IEL (Fig 5L).

**Fig 6:**
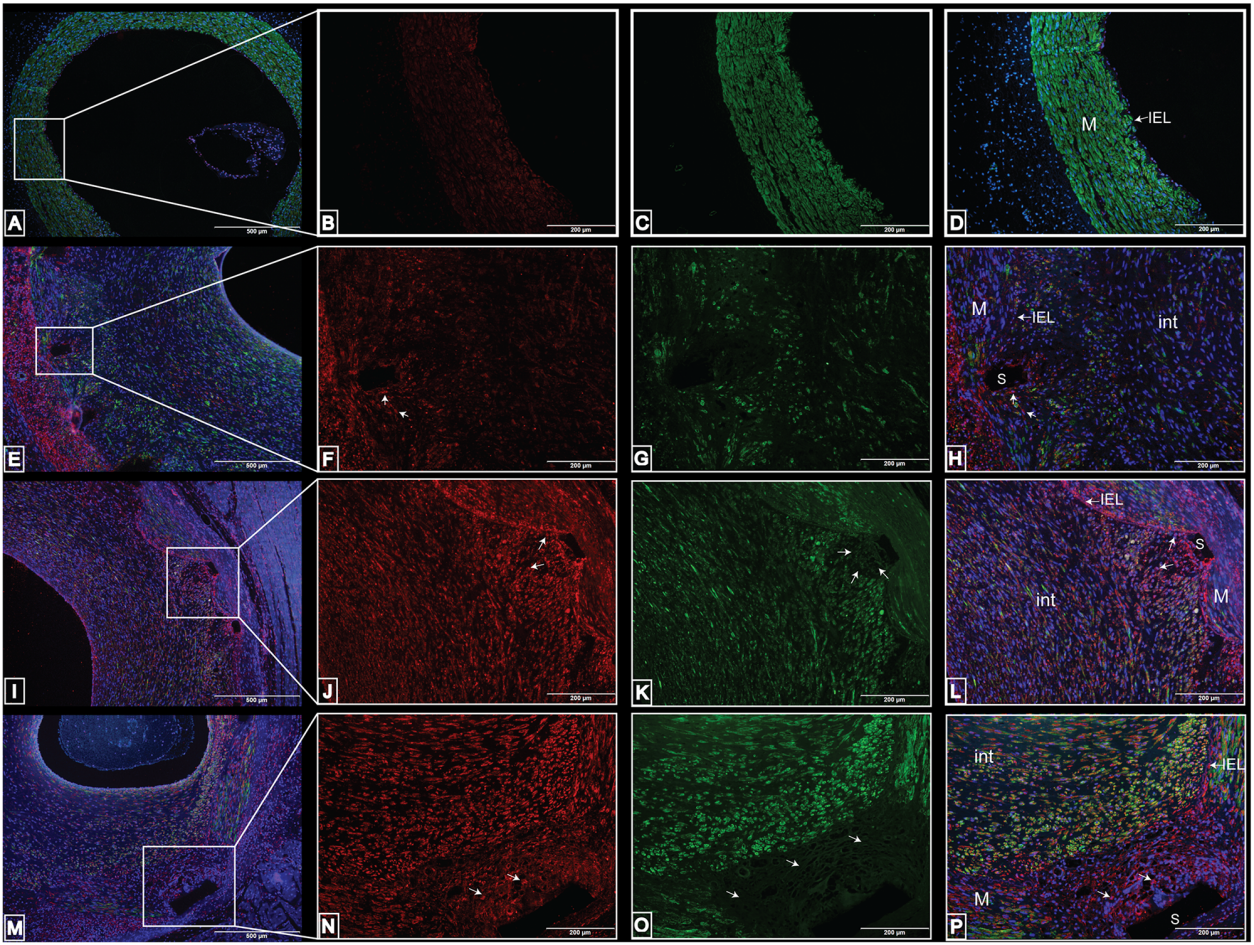
Immunoreactivity of vimentin and alpha-smooth muscle actin in non-stented versus stented swine coronary arteries. Immunostaining in non-stented (A-D) and stented (E-P) swine coronary arteries shows the distribution of vimentin in the media and endothelial cells in the non-stented coronary arteries (B) and the localization of SMA in the smooth muscle cells in the media (C-D). Immunostaining in stented swine coronary arteries with an injury score less than 1 (E-H) shows the immunopositivity of vimentin to cells near the stent struts and the immunoreactivity of SMA to the neointima and media (G-H). Immunostaining in stented swine coronary arteries with an injury score of 1 to 1.5 (I-L) shows the immunopositivity of vimentin to cells near stent struts (J) and the positive reactivity of SMA to the neointima (K-L). Immunostaining in stented swine coronary arteries with an injury score greater than 2 (M-P) shows the immunopositivity of vimentin to the neointimal cells (N) and immunopositivity of SMA to cells in the neointima and media (O-P). These cells have different shaped nuclei (rounded in neointima and elongated in media). These are representative histological slides from N = 3 in each injury score group.

The immunoreactivity of alpha-smooth muscle actin-SMA (green) and vimentin (red) was documented in non-stented and stented coronary arteries (Fig 6). In the non-stented coronary arteries, the vimentin antibody showed immunoreactivity to cells within the media and luminal endothelial cells (Fig 6B), whereas most of the smooth muscle cells within the media expressed SMA (Fig 6C and 6D). In the stented coronary arteries with a mean injury score less than 1, there was immunostaining of vimentin to cells near the stent struts (Fig 6F) and SMA immunostaining within smooth muscle cells in the media and in the neointima (Fig 6G). The majority of the SMA immunostaining was found within the media or in the neointimal region in proximity to the stent struts (Fig 6H). In stented coronary arteries with a mean injury score of 1 to 1.5, more cells near the stent strut showed positive vimentin immunoreactivity (Fig 6J). Immunostaining of SMA was present in two morphologically distinct smooth muscle cells (rounded nuclei in the neointima and elongated nuclei in the media). However, the cells near the stent struts did not react to the SMA antibody (Fig 6K and 6L).

In the stented coronary arteries with a mean injury score greater than 2, immunostaining of vimentin was present in the neointimal cells and in cells next to the stent struts (Fig 6N), and immunostaining of SMA was detected in smooth muscle cells of the media and neointima but was completely absent from neointimal cells near the stent struts (Fig 6O and 6P). The smooth muscle cells in close proximity to the IEL had distinctively rounded nuclei in comparison with the oval shaped nuclei of the smooth muscle cells in the media. The immunoreactivity of the SMA antibody was distributed throughout the media in the non-stented coronary arteries (Fig 6A, 6C and 6D) and the media and neointima of the stented coronary arteries with a mean injury score less than 2 (Fig 6E, 6G, 6I and 6K). However, there was an absence of SMA expression in cells within the neointima (in that portion of maximum neointima thickness) in stented coronary arteries with a mean injury score greater than 2 (Fig 6M).

**Fig 7:**
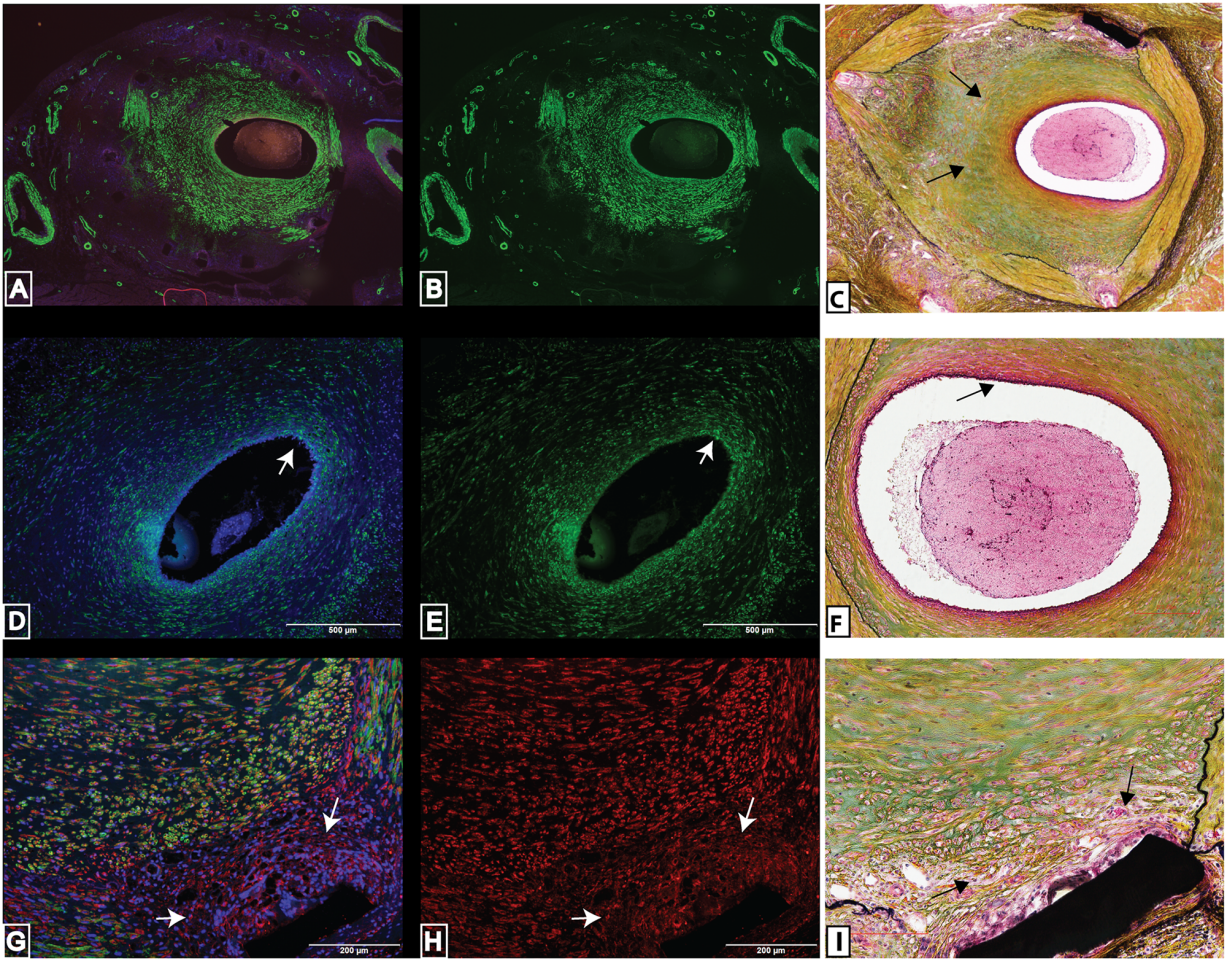
Summary of smooth muscle cell response to stent injury in coronary arteries. Alpha-smooth muscle actin immunoreactivity was found solely in the luminal side of the neointima and absent from areas surrounding stent struts and external elastic region of neointima (A-B) in stented coronary arteries with an injury score greater than 2. The neointima region that is positive to alpha-smooth muscle actin also stained green in the Movat’s pentachrome stain, indicating the presence of proteoglycans (C). Spindle shaped smooth muscle cells that are positive to alpha-smooth muscle actin are concentrated around the lumen of stented coronary arteries with an injury score greater than 2 (D-E) and those cells stained red in Movat’s pentachrome stain indicating the presence of muscle cells (F). Alpha-smooth muscle actin is absent from neointimal cells surrounding stent struts in stented coronaries with an injury score greater than 2 (G), however, those cells are positive for a synthetic smooth muscle cell marker, vimentin (H). There was a lack of green stain (proteoglycans) and possible necrotic cells surrounding the stent strut (I). These are representative histological slides from N = 3 in each injury score group.

In the stented arteries with an injury score greater than 2, only the luminal portion of the neointima was positive for SMA (Fig 7A and 7B), and this region of the neointima was stained green after Movat’s staining, revealing this area to be composed of proteoglycans secreted by smooth muscle cells (Fig 7C). The media and neointimal regions next to the stent struts that were not positive to SMA were composed of collagen (media-yellow stain from Movat’s) or lacked any stain due to the paucity of cells (near the stent struts). The smooth muscle cells surrounding the lumen were composed of spindle shaped cells that were highly concentrated around the lumen (Fig 7D and 7E), also reflected in the dark red color that surrounds the lumen as indicated by the Movat’s stain (Fig 7F). We did find differently shaped smooth muscle cell nuclei (elongated nuclei versus rounded nuclei) in the media versus the neointima, respectively. The neointimal regions next to the stent struts that were not positive to SMA were positive for vimentin, (a synthetic smooth muscle cell marker) and elliptical in shape (Fig 7G and 7H).

## Discussion

In this study, we found a correlation between percent area stenosis and injury score (r = 0.586 p = 0.05), although this correlation was not as significant as that reported by Schwartz et al. (r = 0.74 p <0.001) [2]. Similar to Schwartz et al. [7] and Indolfi et al. [8], cell proliferation was highly correlated with fragmentation of the IEL and tunica media injury. In the stented coronary arteries with a mean injury score greater than 2, (indicating damage to the IEL and media) the immunoreactivity of Ki67 and HMGB1 was greater in cells surrounding stent struts that had fractured the IEL. The elastin membrane may influence aspects of neointimal formation as evidence exists that the IEL may act as a barrier against macromolecules from the lumen [9]. If the IEL barrier fractures and an inflammatory response activates macrophages which produce elastin dissolving macromolecules [10], further damage to the integrity of the IEL may occur.

Arterial injury alone, instead of diet, may be sufficient to influence inflammation, proliferation, neointimal thickening, and eventually restenosis. Swine fed a normal diet that was average in lipid content had stented coronary vessels with proliferative restenosis [7] and no differences were found in the neointimal thickness of stented coronary arteries in swine fed either a cholesterol-raising diet or a normal diet [11]. Proliferation of smooth muscle cells is possible without a high lipid diet but inflammation-induced macrophage recruitment and plaque formation due to a high lipid diet may exasperate the necrotic or apoptotic response in cells near the stent struts.

HMGB1 may act as a pro-inflammatory stimulant that induces the migration of smooth muscle cells [12], especially after injury. HMGB1 is normally expressed in human aortic intimal smooth muscle cells [12], and is also released from necrotic [13] or apoptotic cells [14]. Hence the HMGB1+ cells near the stent struts may indicate tissue damage and the HMGB1+ smooth muscle cells near the fragmented IEL may be responding to cellular HMGB1 by migrating into the neointima. Smooth muscle cells may be migrating and possibly proliferating as HMGB1 was also shown to induce proliferation in smooth muscle cells [15], and inhibiting HMGB1 caused an attenuation to smooth muscle cell proliferation and neointimal formation after mechanical injury [16]. Reducing proliferation and migration of smooth muscle cells may be the key to increasing the life of stents. But, inflammatory cells also play a role in modulating the arterial wall response to stent injury. Restenosis was decreased when inflammatory response and proliferation were suppressed [17].

In the stented arteries with an injury score greater than 2, two different smooth muscle cell phenotypes were detected based on positive immunoreactivity to alpha-smooth muscle actin and vimentin and based on the shape of the nuclei. The cells surrounding the lumen and within the media were spindle shaped and positive for the contractive marker, alpha-smooth muscle actin; whereas cells within the neointima were rounded and positive for the synthetic marker, vimentin. Different smooth muscle cells phenotypes have been reported in human coronaries exposed to bare metal stents [18], with synthetic smooth muscle cells being more frequent. Given the predisposition of elliptical smooth muscle cells to apoptosis following mechanical injury [19–20], and a lack of an eosinophilic (shown by H&E stain) and proteoglycan matrix around these cells indicates extensive mechanical damage to the smooth muscle cells surrounding the stent struts.

## Conclusions

In conclusion, arterial injury caused by stent implantation may be the leading cause of inflammation and proliferation that induces neointimal thickness and percent area stenosis in coronary arteries of the swine model. Furthermore, the distribution of actively proliferating cells and contractile smooth muscle cells may depend on the degree of arterial injury.
